# Phytochemical-assisted green synthesis of CuFeO_*x*_ nano-rose electrocatalysts for oxygen evolution reaction in alkaline media†

**DOI:** 10.1039/d3ra02512h

**Published:** 2023-06-23

**Authors:** D. K. Sarkar, V. Selvanathan, M. Mottakin, A. K. Mahmud Hasan, Md. Ariful Islam, Hamad Almohamadi, Nabeel H. Alharthi, Md. Akhtaruzzaman

**Affiliations:** a Solar Energy Research Institute, Universiti Kebangsaan Malaysia Bangi Selangor Darul Ehsan 43600 Malaysia akhtar@ukm.edu.my vidhya@ukm.edu.my; b Department of Applied Chemistry and Chemical Engineering, Rajshahi University Rajshahi-6205 Bangladesh; c Institute of Sustainable Energy, Universiti Tenaga Nasional (The Energy University) Jalan Ikram-Uniten Kajang 43000 Selangor Malaysia; d Department of Applied Chemistry and Chemical Engineering, Bangabandhu Sheikh Mujibur Rahman Science and Technology University Gopalganj-8100 Bangladesh; e Department of Chemical Engineering, Faculty of Engineering, Islamic University of Madinah Madinah Saudi Arabia hha@iu.edu.sa; f Department of Mechanical Engineering, Faculty of Engineering, Islamic University of Madinah Madinah Saudi Arabia; g Department of Mechanical Engineering, College of Engineering, King Saud University Riyadh 11421 Saudi Arabia; h Graduate School of Pure and Applied Sciences, University of Tsukuba Tsukuba Ibaraki 305-8573 Japan

## Abstract

This study represents a green synthesis method for fabricating an oxygen evolution reaction (OER) electrode by depositing two-dimensional CuFeO_*x*_ on nickel foam (NF). Two-dimensional CuFeO_*x*_ was deposited on NF using *in situ* hydrothermal synthesis in the presence of *Aloe vera* extract. This phytochemical-assisted synthesis of CuFeO_*x*_ resulted in a unique nano-rose-like morphology (petal diameter 30–70 nm), which significantly improved the electrochemical surface area of the electrode. The synthesized electrode was analyzed for its OER electrocatalytic activity and it was observed that using 75% *Aloe vera* extract in the phytochemical-assisted synthesis of CuFeO_*x*_ resulted in improved OER electrocatalytic performance by attaining an overpotential of 310 mV for 50 mA cm^−2^ and 410 mV for 100 mA cm^−2^. The electrode also sustained robust stability throughout the 50 h of chronopotentiometry studies under alkaline electrolyte conditions, demonstrating its potential as an efficient OER electrode material. This study highlights the promising use of *Aloe vera* extract as a green and cost-effective way to synthesize efficient OER electrode materials.

## Abstract 2

Interconnected nanosheets and higher Fe and Ni content improved OER kinetics and lower the overpotential of the CuFeO_*x*_ catalyst to only 410 mV to reach 100 mA cm^−2^. The catalysts exhibited excellent stability for more than 50 hours.

## Graphical abstract

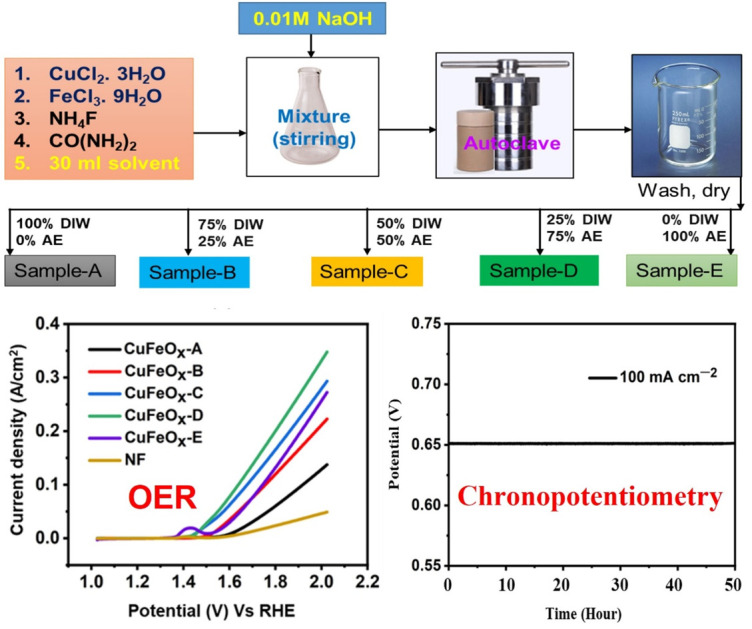

## 1. Introduction

Fossil fuels, such as coal, oil, and natural gas, make up 81% of our existing energy system, which raises issues because of their quick depletion and emissions.^1^ On the other hand, conventional renewable energy sources like solar, wind, hydro, and geothermal are not convenient due to their low energy density and local production issues.^2^ Instead of using conventional renewable energy sources for transportation fuel, they could be used for producing green hydrogen through water electrolysis. Green hydrogen is being treated as the alternative to carbon-based fuel and the fuel of the future due to its lack of resource constraints and emissions concerns.^3^ Moreover, hydrogen has the highest energy density of any fuel in gravimetric quantity.^4,5^ The most abundant component in the world is water which can be split into hydrogen and oxygen for use as fuel. When burned, hydrogen produces only water, making it a clean and sustainable energy source.^6^ This game-changer and sustainable technology could not be explored due to some drawbacks in the production of hydrogen, its storage, and its transportation system.

Water splitting (WS) takes place through the OER and hydrogen evolution reaction (HER), where the OER part is most challenging because of its complexity and sluggish chemical kinetics process.^5^ The practically higher overpotential (OP) of the OER step consumes a large amount of energy for the operation, which is related to the high production cost of hydrogen. Without catalytic support, the whole production is not economically viable. Therefore, searching for suitable electrocatalysts is an attractive point to the researchers. The best-performing electrocatalysts (ECs) are noble metal-based materials such as RuO_2_, IrO_2_,^7,8^ AgPtO_*x*_ for OER^9^ and Pt, Au, Pd, Ag, AgPtO_*x*_, AgAuO, AgPdO_*x*_, *etc.* for HER.^10–12^ The abovementioned ECs regarded as state-of-the-art and exhibit extraordinary performance in catalytic water splitting. But their extreme scarcity due to less natural resources, high cost, and inferior stability make a barrier to use them in large-scale hydrogen production.^6^ Nowadays, various earth-abundant transitional metals like Cu, Fe, Co, Ni, *etc.*^13–18^ with their alloys, oxides,^19–21^ chalcogenides,^14^ oxyhydroxides, hydroxides,^18,21^ phosphides,^22^ metal organic framework (MOF)^23^ have been reported as very much promising ECs owing to their performance and excellent stability. Recent reports discuss significant advancements in modifying catalyst surfaces and developing their morphology to yield electrocatalyst with expanded surface area.^24,25^ Generally, the use of organic molecules as capping and complexing agents are commonly employed strategies to achieve interesting morphologies of electrocatalysts directly grown on conductive substrates.^26–28^ The decoration of foreign materials in such nanostructures play a vital role in the OER catalysis process.^29^ Introducing inexpensive and easily accessible transitional metals (TMs) into a multiphase system is considered more beneficial for creating more active points that enhance electrical conductivity and ultimately lead to improved performance.^21,30,31^

Particularly, the combination of copper and iron for electrocatalytic material have shown encouraging results. In 2020, Xu *et al.* developed selenium enriched copper-iron selenide on copper foam as highly active catalysts for oxygen evolution through the optimization of surface morphology.^32^ The unique composition recorded ultralow overpotential of 200 mV at 10 mA cm^−2^. Inspired by these findings, in this work, a combination of copper-iron oxides synthesized *via* hydrothermal in different chemical environment is explored for catalyzing the OER. Some of the commonly used complexing agent used in hydrothermal synthesis of binder-free metal oxides-based electrocatalyst include urea, ammonium fluoride (NH_4_F), cetyltrimethylammonium bromide (CTAB), sodium dodecyl sulfate (STS) and polyvinylpyrrolidone (PVP).^33–37^ However, in line with the recent awareness towards green chemistry, these synthetic additives can be substituted with green complexing agent derived from phytochemicals which in fact have been used commonly in synthesis of metal oxide nanoparticles. The selection of plant extract utilized during the synthesis procedure plays a vital role in determining how ions are reduced, as well as in capping and stabilizing metal oxide nanostructures. Ultimately, this choice influences the overall morphology of the nanostructure.^38,39^

Therefore, in this work, the potential of using *Aloe vera* extract complexing agent for efficient morphology engineering is studied. *Aloe vera* gel primarily consists of water and polysaccharides like pectins, cellulose, hemicellulose, glucomannan, and acemannan. Additionally, the aloe latex contains hydroxyanthracenic derivatives, anthraquinone glycosides, and emodin.^40,41^ The unique chemical cocktail in the *Aloe vera* extract is expected to facilitate the formation of distinct nanostructured morphology that will boost its electrocatalytic activity. The catalysts were prepared through *in situ* hydrothermal and solvothermal processes, and five samples were produced, namely CuFeO_*x*_-A, CuFeO_*x*_-B, CuFeO_*x*_-C, CuFeO_*x*_-D, and CuFeO_*x*_-E, which varied based on the percentage of *Aloe vera* extract used. To evaluate the impact of the natural complexing agent on the catalysts' performance, the samples underwent characterization using X-ray diffraction (XRD), field effect scanning electron microscopy (FESEM), energy dispersive X-ray (EDX), and the necessary electrochemical tests. The study provides valuable information on the use of green synthesis of bimetallic heterostructure electrocatalysts for efficient water splitting.

## 2. Materials and methods

### 2.1 Chemicals

Chemicals, CuCl_2_·3H_2_O, FeCl_3_·9H_2_O, NH_4_F, CO(NH_2_)_2_, C_2_H_5_OH, KOH, and dry starch, including Nickle Foam (NF) were supplied from Sigma-Aldrich, Chemical Reagent Co. Ltd. Sdn. Bhd. Malaysia, and all purchased chemicals were used in the experiment without further purification.

## 3. Preparation of copper iron oxide (CuFeO_*x*_) electrocatalyst

### 3.1 Preparation of solvent

Firstly, *Aloe vera* stems were cleaned with deionized (DI) water, dried, and sliced. In a glass beaker, 15 g *Aloe vera* slices were placed in 100 ml ethanol and swirled for 1 hour at 40–45 °C on a hot plate. The mixture was then filtered and kept in the refrigerator to be utilized as a co-solvent and complexing agents in synthesis. The whole solvent preparation process was followed by the previous study.^42,43^

### 3.2 Materials synthesis

For the synthesis of Cu, Fe-based oxide OER electrocatalysts on NF, a modified hydrothermal method and solvothermal methods were employed. The AE and DI water mixture was taken as a solvent in this deposition process. DI water and phytochemicals in AE which acted as complexing or capping agents and precipitators for producing metal oxides at the high vapor pressure of the mixed solvent.

### 3.3 Deposition of CuFeO_*x*_-based catalyst electrodes

**Fig. 1:**
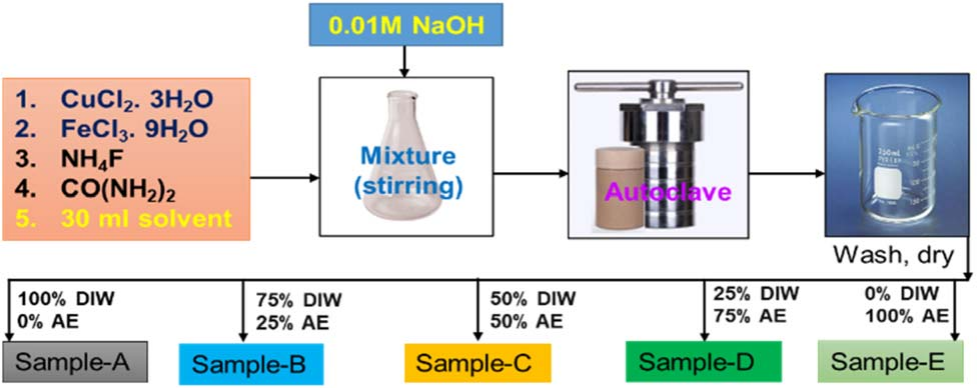
Schematic diagram of the catalyst preparation process.

In the deposition of CuFeO_*x*_-based electrocatalysts, five samples were grown on NF by simple hydrothermal methods. First, the precursors were produced by mixing 2 mmol each of CuCl_2_·3H_2_O and FeCl_3_·9H_2_O with 4 mmol NH_4_F, 6 mmol CO(NH_2_)_2,_ and 30 ml DI water as solvent. All of the materials were placed into a 250 ml conical flask and stirred vigorously for 50 minutes. Then, the pH of the mixture was changed to 9 by slowly adding 1 M NaOH solution drop by drop. Finally, it was moved into a 50 ml stainless steel autoclave lined with Teflon. As usual NF (1 × 2 cm^2^) was cleaned by following the previously applied procedure,^44,45^ and the cleaned NF was placed in the autoclave precursor solution before being sealed. To finish hydrothermal coating, the sealed autoclave was heated at 130 °C for 6 hours. After cooling at ambient temperature, the sample was placed in a beaker and rinsed 3–4 times with DI water and ethanol. It was then dried for one hour at 100 °C. The same procedures were followed for the 2nd, 3rd, 4th and 5th samples with the exception of replacing 30 ml of DI water solvent with AE at 25%, 50%, 75%, and 100% by volume. The obtained samples were named CuFeO_*x*_-A, CuFeO_*x*_-B, CuFeO_*x*_-C, CuFeO_*x*_-D, and CuFeO_*x*_-E, respectively. The preparation process of the catalyst electrodes is simplified by the following Fig. 1.

### 3.4 Structural and morphological characterization

An X-ray diffractometer was used to determine the crystal structure of the produced samples (Bruker AXS D8 ADVANCE) at a scan rate of 2° per minute. The morphology and nanostructure with elemental composition and mapping were studied by FESEM and EDX. The above characterizations were repeated for the best-performing sample after the electrochemical and chronopotentiometric stability test.

### 3.5 Electrochemical characterization

Using a computerized Metrohm Autolab workstation, electrochemical measurements were performed (Metrohm, Herisau, Switzerland) in a three-electrode system using a 1.0 M KOH solution as an electrolyte. The synthesized samples were used as working electrodes, and a platinum (Pt) thin plate and a saturated silver–silver chloride salt solution (Ag/AgCl) electrode (SAACE) were employed as counter electrodes and reference electrodes, respectively. The recorded potential data were converted into the reversible hydrogen electrode (RHE) by the relation as follows,*E*_RHE_ = *E*_Ag/AgCl_ + 0.695 + 0.0591 × pH

OER performance was examined using linear sweep voltammetry (LSV) at 2 mV s^−1^. Before final LSV measurements, the electrodes were scanned from 0.3 to 0.8 V (*vs.* SAACE) 20 mV S^−1^ until the electrode became stabilized. Tafel slopes were calculated from the nearest static LSV data. Overpotential (OP) is derived from the recorded LSV results based on the equation below.OP = *E*_RHE_ − 1.23 V

The electrochemical active surface area (ECSA) was derived based on the double-layer capacitance (*C*_dl_) by conducting a series of CV at the scan rate of 30 to 80 mV s^−1^ in a non-faradaic region (0.157–0.357 V *vs.* SAACE). The electrochemical impedance spectra (EIS) were evaluated within the frequency range from 100 mHz to 100 kHz. Finally, the stability measurement test was carried out by chronopotentiometry (CP) at the applied voltage of 0.35 V to achieve a constant current density (*J*) of 1.0 mA cm^−2^.

## 4. Results and discussion

### 4.1 XRD analysis of CuFeO_*x*_-based electrode samples

**Fig. 2:**
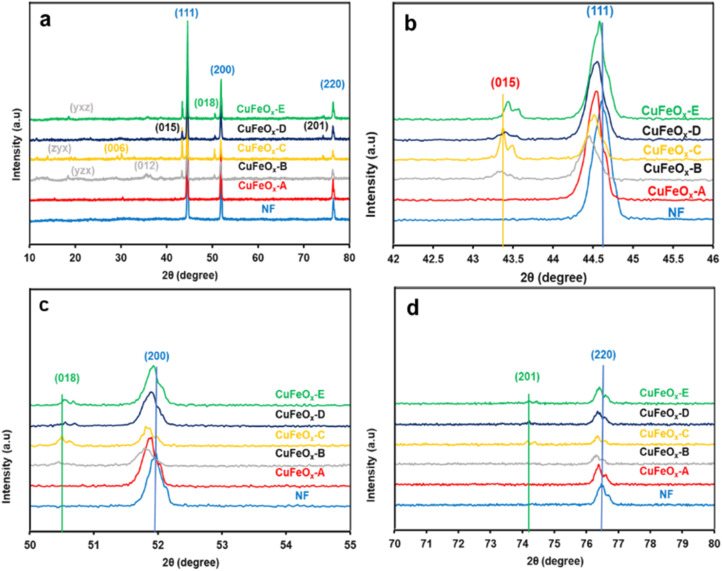
XRD pattern of the synthesized CuFeO_*x*_-based electrode samples and NF; (a) from 10° to 80°, (b) from 42° to 46°, (c) from 50° to 55°, and (d) from 70° to 80°.

XRD is a strong characterization tool to determine the crystalline structure and material phases formed in the synthesized samples. The XRD patterns of all synthesized electrode samples are depicted in Fig. 2. The most prominent peaks of Nickel foam (NF) are found at 2*θ* angles of 44.65°, 51.92°, and 76.88° with corresponding crystal planes (111), (200), and (220) have been shown in Fig. 2(a).^46^Fig. 2(a) also shows some relatively small peaks on the indexed plans (006), (012), (015), (018), and (201) at the 2*θ* value of 30.23°, 35.66°, 43.44°, 50.68°, and 74.22° respectively.^47–50^

These all are assigned signals of CuFeO_*x*_-based material (CuFeO_2_, Ni:CuFeO_2_, NiFeO_2_, *etc.*) on the NF, but their peak intensity is very diffused due to the presence of very high intensities of NF crystallinity.^51^ In the preparation process, DI water and *Aloe vera*–ethanol extract and their mixture are used as the solvent. Phytochemicals in *Aloe vera*–ethanol extract (AE) influences the crystallinity and material composition of the prepared catalyst samples in presence of solvent polarity. In Fig. 2(b), it can be found that the highest peak signal of all (CuFeO_*x*_-A, CuFeO_*x*_-B, CuFeO_*x*_-C, CuFeO_*x*_-D, and CuFeO_*x*_-E) samples on the plan (111) has shifted toward a lower diffraction angle from pure NF's peak. The main reasons for the peak shifting are the development of larger crystalline defects and the diffusion of Ni^2+^/Ni^3+^ in CuFeO_*x*_ phases.^52,53^ Similar cases have been seen for the peaks in indexed planes (200), and (220) assigning Fig. 2(c) and (d). The characteristic XRD signals of CuFeO_*x*_-based delafossite crystal are observed in plans (015), (018), and (012) which are too defused compared to the peaks in plan (111) as shown in Fig. 2(b)–(d). The diffraction peaks of the agglomerated nanosheets (as seen in the FESEM image) of the CuFeO_*x*_-C sample are clearly distinguishable from those of the other samples, except for NF. The lower XRD intensity seen in samples B and C in Fig. 2. Lower crystallinity, smaller particle size, the presence of structural defects or impurities, and various crystallographic phases are all potential causes. These variables may affect the overall crystalline order, which can result in decreased XRD intensity. Besides these, some other unidentified diffraction peaks in planes (*yzx*), (*zyx*), (*yxz*) are found in the lower 2*θ* angle which required more studies. The overall XRD study of the prepared catalyst materials illustrates that the deposited films are very thin (low peak intensity) with mostly polycrystalline, and multiphases.

### 4.2 Field emission scanning electron microscopy (FESEM)

**Fig. 3:**
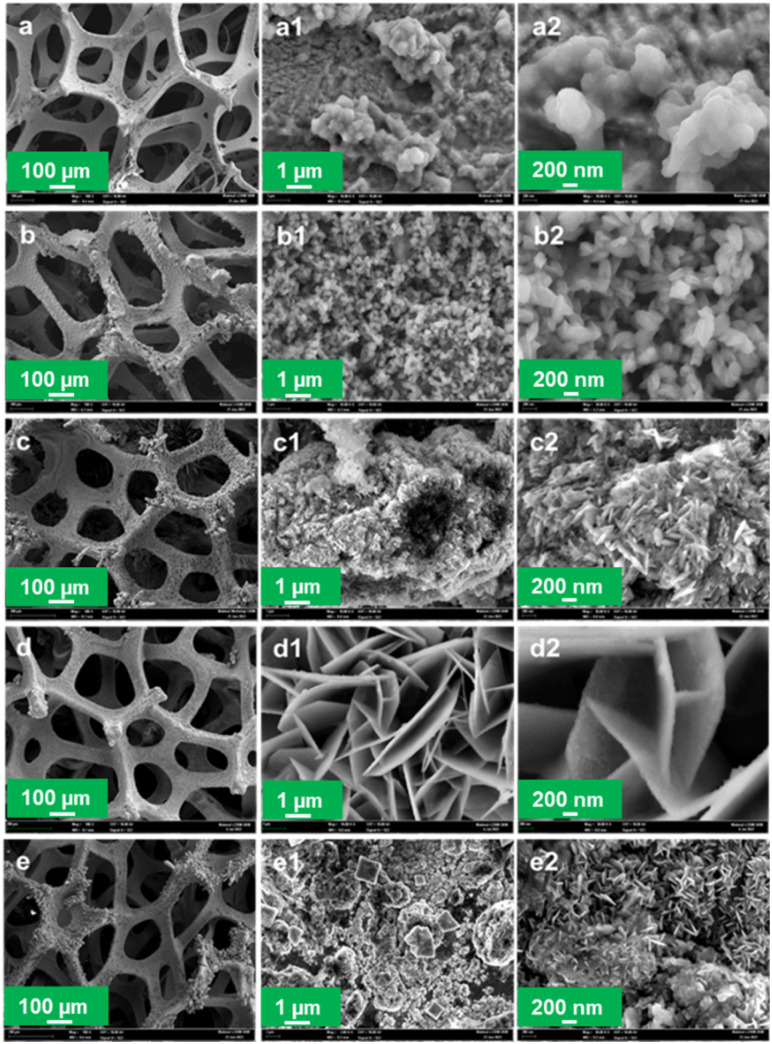
FESEM images of the prepared electrode samples at different magnifications; (a, a1 and a2) CuFeO_*x*_-A, (b, b1 and b2) CuFeO_*x*_-B, (c, c1 and c2) CuFeO_*x*_-C, (d, d1 and d2) CuFeO_*x*_-D, and (e, e1 and e2) CuFeO_*x*_-E.

The morphology and nanostructure of the solvothermally deposited CuFeO_*x*_-based OER electrocatalysts on NF are characterized by FESEM. The FESEM images of the prepared samples have been illustrated in Fig. 3 with different magnifications such as a, a1, and a2 representing the images of the same sample CuFeO_*x*_-A. Images of the sample, CuFeO_*x*_-B are represented by b, b1, and b2. Similarly, the image series of 1c, 1d, and 1e represent the samples CuFeO_*x*_-C, CuFeO_*x*_-D, and CuFeO_*x*_-E, respectively.

The FESEM images in the series shown in Fig. 3(d) illustrate that numerous highly porous interconnected nanosheets have grown vertically on the 3D NF. The diameter of the nanosheets is 30–70 nm. The vertically linked nanosheets have made funnel-types of hollow space insides themselves. As a result, electrode–electrolyte contact surface area increases many folds that enhance the sluggish OER kinetics leading to the lowest OP for WS. A solvent mixture of 25% DI water and 75% AE was used in the synthesis process, where the phytochemicals in AE played the most effective role in creating the optimal composition and morphology of the product. It may be hypothesised that the particular solvent composition has an impact on the rose-like morphology seen with a 75% AE and 25% DIW solvent combination in the FESEM investigation. The formation of unique patterns may result from the favourable nucleation and growth circumstances this particular solvent ratio. The rose-like shape formation may be greatly influenced by the variable surface tensions, reaction rates, and templating effects caused by the solvent combination. Separately, 25% and 50% AE were mixed with DIW and were applied to the samples CuFeO_*x*_-B, and CuFeO_*x*_-C. In both cases, nanostructures have changed distinctly seen in Fig. 3(b) and (c) but activities are limited for smaller active areas due to the large aggregation of nanoparticles in sample CuFeO_*x*_-B, and that of solid nanosheets in the sample CuFeO_*x*_-C. Their OER performances, as shown in Table 1, are higher than those of samples CuFeO_*x*_-A and CuFeO_*x*_-E, in which no mixed solvent was used. The attributed OP and relevant nano structuring morphology of the FESEM study ensured that the chemicals in AE explored their best activities by the polarity of the solvent. The exact mechanism of the mixed solvent demands extensive investigation of experiments.

**Table 1:** EDS results and overpotential of the samples^a^

Sample	% (DIW + AE)	% Ni	% Cu	% Fe	% O	(OP)_50_	(OP)_100_
CuFeO_*x*_-A	100 + 0	8	69	1.7	22	530	690
CuFeO_*x*_-B	75 + 25	25	53	2.8	18	400	530
CuFeO_*x*_-C	50 + 50	56	22	3	19	340	450
CuFeO_*x*_-D	25 + 75	35	24	18	22	310	410
CuFeO_*x*_-E	0 + 100	25	53	1	16	420	510

a Elemental composition of synthesized electrode samples based on EDS and OP for a *J* of 50 mA cm^−2^ and 100 mA cm^−2^.

### 4.3 Elemental composition

**Fig. 4:**
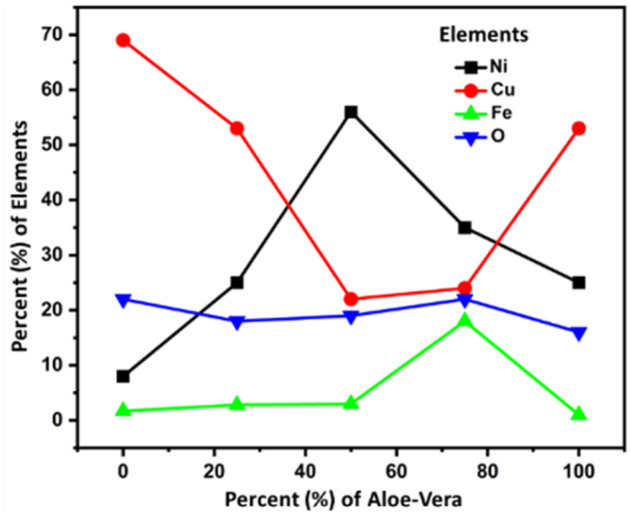
A comparison plot of the elements found through EDX analysis.

The energy dispersive spectroscopy (EDS) results of the samples in Fig. 4 are summarized in Table 1 with the solvent composition and OER overpotential of prepared catalyst electrodes.

Table 1 shows that sample CuFeO_*x*_-D with 75% AE is shown to be the best electrocatalyst. Based on the elemental compositions presented in Fig. 4, it is evident that the composition of solvent plays a vital role in the elemental content of the samples. The samples were prepared in different solvent medium comprising of various amounts of water and ethanol based-*Aloe vera* extract. It is evident that as the solvent content is increased from 0–75% *Aloe vera* extract, the composition of iron content increases and copper content decreases in the sample, reaching 18% of iron and 24% copper in CuFeO_*x*_-D. Realizing that the composition of oxygen remain fairly consistent for all the samples, it is proposed that in samples CuFeO_*x*_-A, CuFeO_*x*_-B and CuFeO_*x*_-D, the significantly high copper percentages indicate formation of copper rich-CuFeO_*x*_. In CuFeO_*x*_-C and CuFeO_*x*_-D, the percentages of copper and iron content in comparison to oxygen suggests the formation of CuFeO_*x*_ with ideal stoichiometry which favours catalytic properties. In addition to the morphological property, the synergistic effect of both copper and iron in CuFeO_*x*_-D assist to improve the overall catalytic performance of the sample. At 100% *Aloe vera* extract, the amount of iron dropped again significantly, indicating that in the absence of water and presence of only *Aloe vera*, the incorporation of iron into copper iron oxide is not favoured. We suspect that the higher viscosity of solvent comprising only *Aloe vera* extract as well as the presence of ethanol as the primary solvent in CuFeO_*x*_-E is not conducive for the uniform formation of copper iron oxide on nickel foam substrate. The nickel content in the samples mainly originated from the nickel foam substrate and thus the higher percentages of nickel in CuFeO_*x*_-C is indicative of the formation of a thin layer of copper iron oxide layer on the nickel foam surface and possibility of exposed nickel foam surface. Fig. S1† displays the EDX spectra of the prepared samples.

With EDX mapping, it is possible to map a sample's elemental distribution. It focused electron beam across the sample and detecting the distinctive X-rays given off by the elements. The distribution of each element throughout the sample surface is displayed on the resultant map.

**Fig. 5:**
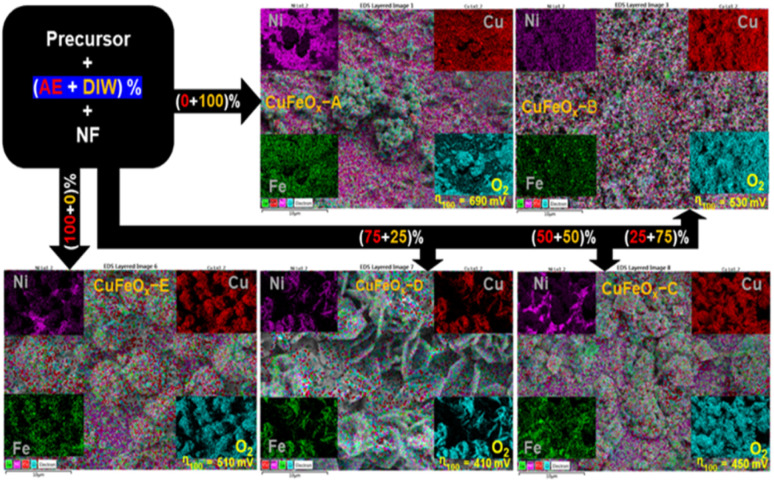
Elemental mapping and performance of synthesized catalyst samples.

According to Fig. 5, the CuFeO_*x*_-based electrocatalyst seems to have a regular distribution of components over the surface of the electrode. The distribution of Cu, Fe, and O on the surface appears to be uniform according to the elemental map, which means that there are no obvious regions of high or low element concentration. The distribution of elements is found to be most homogeneous but the metal content in each sample is dominated by the amount of AE present in the mixed solvent. In addition, the electrode's surface seems rough rather than smooth, indicating that the electrode has a larger surface area. Electrocatalysts with greater surface areas are preferred because they can offer more active locations where catalytic reactions can take place. This study enhanced the structural integrity and porosity of the electrodes, by depositing very thin dendritic and porous Fe, Ni, and Cu nanostructures on the NF. This was accomplished by adopting a quick and easy *in situ* solvothermal process in which O_2_ bubbles serve as templates for the development of pores. Here, four different catalysts based on AE phytochemicals were prepared on high-surface-area support, and in each case, they should generate larger double-layer capacitances, indicating that the active sites are more accessible.

### 4.4 X-ray photoelectron spectroscopy (XPS)

**Fig. 6:**
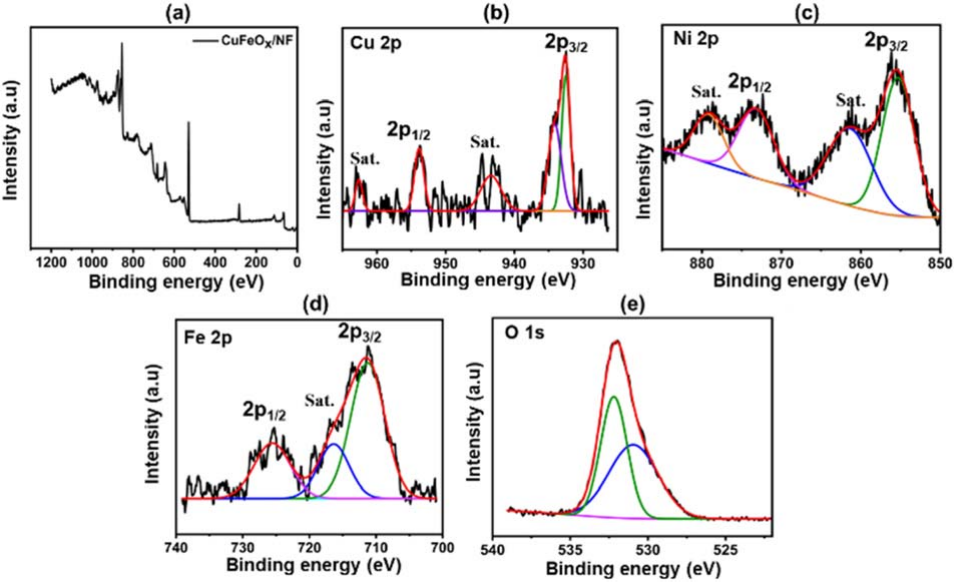
XPS analysis of CuFeO_*x*_-D sample; (a) CuFeO_*x*_-D survey peak, (b) Cu 2p, (c) Ni 2p, (d) Fe 2p, (e) O 1s.

The CuFeO_*x*_-D sample's XPS peak is shown in Fig. 6(a). The peaks of Cu 2p_3/2_ at 932.52 and 934.2 eV, as seen in Fig. 6(b), point to the presence of metallic Cu^o^ (and/or Cu^1+^) and Cu^2+^ species. At 943.6 eV and 962.72 eV, weak-intensity satellite peaks were also seen, pointing to the presence of Cu^+^ on the sample's surface.^54^ During the hydrothermal process, the binding energy of Ni 2p_3/2_ at 855.36 eV and Ni 2p_1/2_ at 873.19 eV potentially originates from the NF, which indicates the presence of Ni^2+^ in the sample accompanied by an enhanced electron density.^55^ The peaks observed at 725.66 eV and 711.21 eV, which correspond to Fe 2p_1/2_ and Fe 2p_3/2_, respectively, provide evidence for the presence of Fe^3+^ in the form of Fe_2_O_3_.^56^ The sample's oxygen lattice (O_Latt_) exhibits a peak at 530.96 eV, while a second peak at 532.17 eV is attributed to adsorbed oxygen (O_Ads_). It is likely that this second component is associated with the diffusion of oxygen atoms into the bulk material.^57^ The addition of copper promotes the incorporation of more O_Latt_. Consequently, the higher peak area of O_Latt_/O_Ads_ becomes significantly influential in driving the catalytic process.^58^

### 4.5 Performances of the prepared electrodes as OER catalyst

**Fig. 7:**
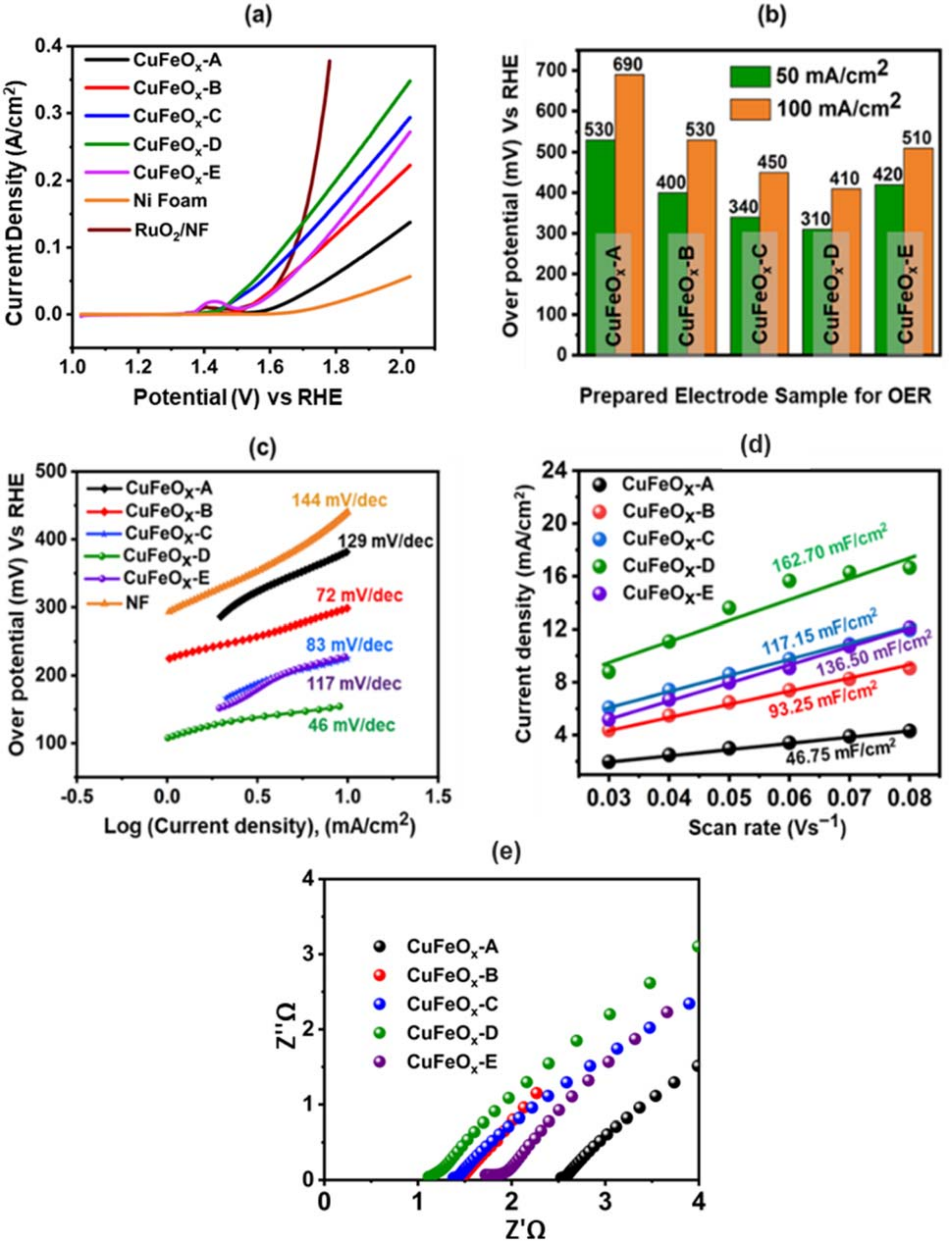
(a) Polarization curve of prepared electrode samples at 1 M KOH for OER; (b) OP at the *J* of 100 mA cm^−2^; (c) Tafel's slope; (d) *C*_dl_ plots of different scan rates *vs. J*; (e) Nyquist plots of the electrodes.

The OER catalytic performances of all prepared samples are evaluated in 1.0 M KOH. The LSV curves are shown in Fig. 7(a). The OER performance of NF is poorer than other electrodes, and the sample CuFeO_*x*_-D has shown the best performance. CuFeO_*x*_-D has the lowest OP of 310 mV and 410 mV to produce at *J* of 50 mA cm^−2^ and 100 mA cm^−2^, respectively. The FESEM image series 3(d) shows the sample CuFeO_*x*_-D with a morphology of interconnected nanosheets, and this type of morphology is responsible for the lowest OP. Besides its morphological features, the higher content of Fe and Ni may be responsible for enhanced performance as seen in EDX analysis (Fig. 4). In terms of catalysis, Fe, and Ni both are superior to Cu.^59,60^ Small peak is observed in LSV for the sample CuFeO_*x*_-B and CuFeO_*x*_-E due to the oxidation of Ni into higher valency state.^59,60^ So, the current not only comes from the OER but also from Ni oxidation. Similar catalytic performances of other samples CuFeO_*x*_-A, CuFeO_*x*_-B, CuFeO_*x*_-C, and CuFeO_*x*_-E are shown in Fig. 7(b), where all are inferior to that of CuFeO_*x*_-D with an OP of 690, 530, 450, and 510 mV respectively at *J* of 100 mA cm^−2^. The OP of CuFeO_*x*_-A is the highest among all synthesized electrodes and shows an OP is 530 mV and 690 mV at *J* of 50 mA cm^−2^ and 100 mA cm^−2^, respectively. But the most promising catalytic performance shown by the catalyst CuFeO_*x*_-D. At 100 mA cm^−2^, the overpotential of CuFeO_*x*_-D is lower compared to commercially applied RuO_2_/NF (447 mV at 100 mA cm^−2^). Despite exhibiting superior catalytic activity of RuO_2_ at higher current densities, CuFeO_*x*_ is readily available and inexpensive, making it commercially more viable. The CuFeO_*x*_-D electrochemical catalyst prepared with 25% AE exhibited an excellent performance with an OP of 80 mV at *J* of 10 mA cm^−2^. This result is unprecedented in the previously published literature with Cu–Fe-based materials.^61,62^

**Fig. 8:**
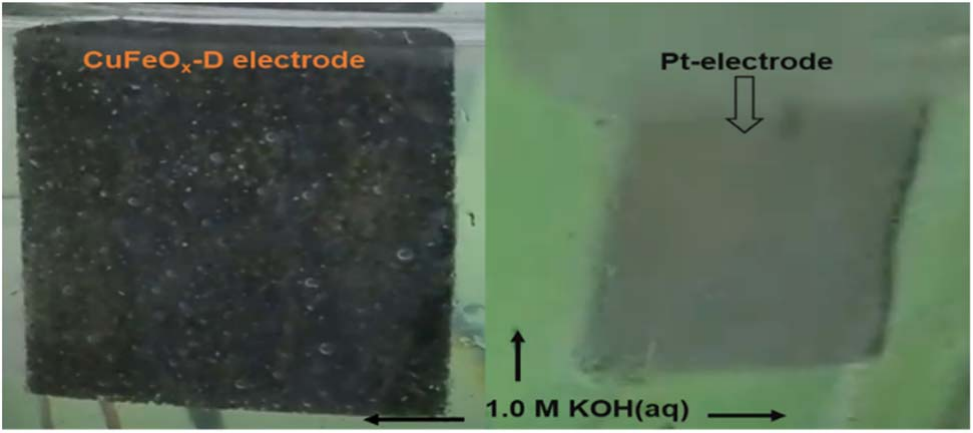
During water splitting in 1.0 M KOH electrolyte; O_2_ evolves at CuFeO_*x*_-D and H_2_ at Pt electrodes.

The electrode CuFeO_*x*_-D has achieved high performance due to its material composition and morphological features. The compositional aspects have been discussed in the EDS section. CuFeO_*x*_-D shows superior performances because of its large surface area and high Fe content, as it is found in FESEM image and EDS results (Table 1). A self-supported conductive network was created by the interconnected nanosheets. This network aided in the transmission of electrons between the catalyst's surface-active sites and the current collector. As the OP increased, a lot of oxygen bubbles were produced on the electrode surface. It prevented the electrolyte from making direct contact with the active sites, which had an impact on how long the reaction would last. As a result, the electrodes' ability to transfer mass and catalysed reactions depended on the release of bubbles. The surface of the Pt electrode contained hydrogen bubbles that had grown and accumulated, as shown in a digital photograph. Moreover, the CuFeO_*x*_-D/NF-produced bubbles quickly disappeared from the electrode surface (Fig. 8), suggesting that the active sites may be quickly re-exposed to the electrolyte. The rapid surface bubble ejection demonstrated that the interconnected nanosheets design of CuFeO_*x*_-D/NF effectively boosted reaction kinetics and facilitated mass transfer.

The Tafel equation (OP = *a* + *b* log *J*) is used to determine the Tafel slopes of the samples, which are displayed in Fig. 7(c). Here, *a* is the intercept-constant and *b* is the Tafel slope. Fig. 7(d) displays the corresponding values of the calculated slopes. The Tafel slope for the sample CuFeO_*x*_-D is the lowest (46 mV dec^−1^) and the respective values of other samples are 129 (CuFeO_*x*_-A), 72 (CuFeO_*x*_-B), 83 (CuFeO_*x*_-C), and 117 mV dec^−1^ (CuFeO_*x*_-E). A higher value of Tafel slope indicates faster electron transport, which in turn suggests a favourable kinetic barrier for the OER.^63^ Therefore, the smaller Tafel slope value denotes the better OER catalytic reaction.

Fig. 7(e) displays the Nyquist plot of the samples, which was derived from the EIS data. EIS is carried out to investigate the ohmic resistance (*R*_ohm_) of the solution/electrode surface and the faradaic charge transfer resistance (*R*_ct_). The semicircle part in the Nyquist plots represents that the faradaic charge transfer process is the rate-determining step for OER.^49^ In the high-frequency range, the intercept of the real axis (*Z*′) with the semicircle section that affects *R*_ohm_ represents the sum of electrode resistance (*R*_e_) and solution resistance (*R*_s_) connected in series (*i.e.*, *R*_ohm_ = *R*_e_ + *R*_s_).^50^Fig. 7(e) shows that a small semicircle with a 0.12 Ω *R*_ct_ is observed. All the prepared samples contain small semicircle and a straight line, indicating better charge transfer through the electrode. CuFeO_*x*_-D has the lowest *R*_ohm_ value of 1.08 Ω cm^−2^ among all the prepared catalyst electrodes, while CuFeO_*x*_-A, CuFeO_*x*_-B, CuFeO_*x*_-C, and CuFeO_*x*_-E have significantly higher values of 2.84, 1.52, 1.43, and 1.91 Ω cm^−2^, respectively, which indicating a quicker reaction kinetics and superior low resistance for the CuFeO_*x*_-D electrode.

**Fig. 9:**
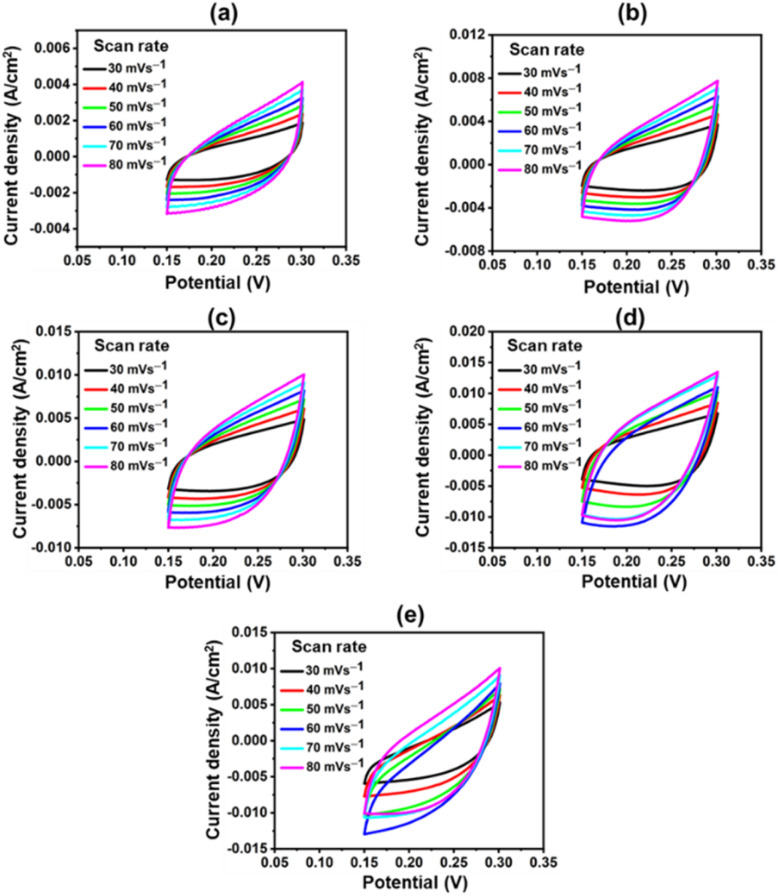
CV polarization curve at various scan rates; (a) CuFeO_*x*_-A, (b) CuFeO_*x*_-B, (c) CuFeO_*x*_-C, (d) CuFeO_*x*_-D, and (e) CuFeO_*x*_-E.

The catalyst's ECSA addresses the intrinsic activity and performance of OER ECs. Data from cyclic voltammetry (CV) tests carried out in the non-faradaic potential range were used to compute the ECSA of the samples based on the double-layer capacitance (*C*_dl_). As shown in Fig. 9, the CV tests were carried out on all samples at potentials ranging from 0.15 V to 0.30 V *vs.* SAACE and at scan speeds of 30, 40, 50, 60, 70, and 80 mV s^−1^. The *C*_dl_ was then calculated from the slope of the linear plot of *J* = (*J*_a_ − *J*_c_, *J*_a_ and *J*_c_ represent anodic and cathodic current densities) at 0.25 V *vs.* SAACE as a function of the sweep rate.^48^ The *C*_dl_ value were used to compute the ECSA. ECSA is equal to *C*_dl_/*C*_s_, where *C*_s_ is the specific capacitance, which is typically equal to 0.040 mF cm^−2^ for metal electrodes in KOH solution. Fig. 9(e) shows the ECSA values of the *C*_dl_ curves of the samples. The calculated ECSA of the samples are 46.75 mF cm^−2^ (CuFeO_*x*_-A), 93.25 mF cm^−2^ (CuFeO_*x*_-B), 117.15 mF cm^−2^ (CuFeO_*x*_-C), 162.7 mF cm^−2^ (CuFeO_*x*_-D), and 136.5 mF cm^−2^ (CuFeO_*x*_-E). The ECSA is directly proportional to active sites so greater ECSA means a larger area of catalyst surface is exposed to reactants species as a result, OER becomes faster.^24^ The performance of the synthesized materials with previously reported similar Cu-based polymetallic OER catalysts are listed in Table 2. In comparison to the literature, the CuFeO_*x*_-D material has a commendable efficiency with overpotential of 175 mV at 10 mA cm^−2^, implying that it could be a promising candidate for electrocatalytic applications. Furthermore, the reported stability of 50 hours demonstrates its robustness and durability under operational conditions.

**Table 2:** Performance of Cu-based polymetallic OER electrocatalysts in WS

No.	Material	*η* (mV)/*J*_sc_ (mA cm^−2^)	Stability	Preparation process	Ref.
1	Cu–NiFe LDH/NF	199/10	24 h	Chemical oxidation	64
2	Cu(OH)_2_:Fe(OH)_3_/CF	365/10	12 h	*In situ* hydrothermal	65
3	NiFe/Cu_2_O NWs/CF	215/10	25 h	*In situ* hydrothermal	66
4	FeCoNiMnCu	280/10	40 h	Cathodic plasma electrolysis deposition	67
5	Cu(OH)_2_–NiFe LDH	283/10	10 h	Unipolar pulse electro-deposition	68
6	CuFe_2_O_4_/NF	340/10	1000 c	Electro-spume	69
7	CaCu_3_Fe_4_O_12_	450/05	11 h	Ion assisted solvothermal	70
8	CuO@Ni/NiFe (OH)_*x*_	230/10	16 h	Chemical oxidation–calcination	71
9	Cu_0.3_Ir_0.7_O_*δ*_	150/100	6000 s	Hydrothermal doping	72
10	RuO_2_·NiO/NF	144/10	72 h	*In situ* grown	73
11	RuO_2_·Ru	172/10	10 000 cycle	Laser ablation	74
12	RuO_2_	320/10	20 h	Electro-chemical	75
13	RuO_2_/CeO_2_	350/10	70 h	*In situ* solution	76
14	Ru–RuO_2_/CNT	210/10	30 h	*In situ* hybrid	77
15	CuFeO_*x*_-D	175/10	50 h	*In situ* solvothermal	This work

### 4.6 Stability

**Fig. 10:**
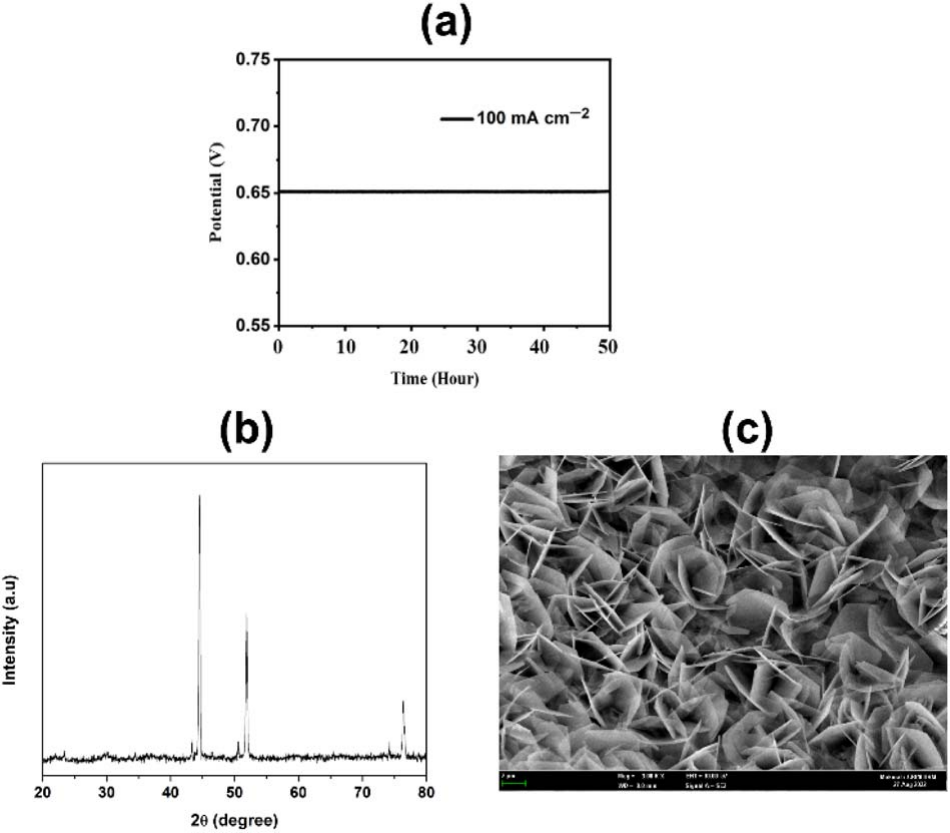
(a) Chronopotentiometry (CP) for the sample CuFeO_*x*_-D at a constant *J* of 100 mA cm^−2^ for 50 hours, (b) XRD diffractogram and (c) FESEM image of CuFeO_*x*_-D after 50 hours stability studies.

The physiochemical stability of electrocatalysts is one of the most important features of their practical application. In this study, the synthesized CuFeO_*x*_-based electrocatalysts were evaluated for their long-time stability. The synthesized electrocatalysts exhibit long-time durability in the assessment test. During the catalytic study, CuFeO_*x*_-D exhibits the lowest OP of 410 mV to reach the *J* of 100 mA cm^−2^. It shows excellent catalytic ability at a constant potential of 0.65 V *vs.* SAACE for 50 hours (Fig. 10(a)). The XRD diffractograms depicted in Fig. 10(b) validate that the diffraction peaks remain largely consistent. The peak position remained the same as before with slight decline in the intensity of all the peaks. Furthermore, Fig. 10(c) demonstrates that the rose-shaped nanostructured catalyst was maintained after the 50 hours stability study, ensuring the robustness of the catalyst in withstanding sustained alkali exposure without severe corrosion. Thus, *in situ* solvothermal deposited CuFeO_*x*_ has shown potential for long time stable catalytic applications in the OER for water splitting.

## 5. Conclusion

The study highlights the potential of using natural complexing agents, such as *Aloe vera*–ethanol extract, in the production of efficient WS electrocatalysts based on CuFeO_*x*_. The use of green synthesis methods for bimetallic heterostructure electrocatalysts can contribute to sustainable and eco-friendly energy production. The phytochemicals in 75% AE are the most effective for high amount Fe content CuFeO_*x*_ based OER catalysts with large ECSA and prospective morphological features. The best electrode CuFeO_*x*_-D shows excellent OER activity, particularly for large current densities. The catalyst performs reasonably well for OER in an alkaline electrolyte. This green synthesized CuFeO_*x*_-D catalyst able to attain cell voltage of 1.75 V and 3.1 V at 10 mA cm^−2^ and 100 mA cm^−2^, respectively. The synthesized electrocatalysts are excellent candidates for real-world use in energy-related domains such as water splitting due to their exceptional stability and catalytic activity.

## Author contributions

D. K Sarkar: writing – original draft and formal analysis; V. Selvanathan: reviewing, editing, revising, and formal analysis; M. Mottakin: drafting figures, validation, formal analysis, writing, and editing; A. K Mahmud Hasan: revision and formal analysis; Mohammod Ariful Islam: revision and editing, Hamad Almohamadi: validation and funding acquisition; Nabeel H. Alharthi: editing and funding acquisition and Md. Akhtaruzzaman: supervision & writing – review & editing.

## Conflicts of interest

There are no conflicts to declare.

## Supplementary Material

RA-013-D3RA02512H-s001
