# Supplementary material for: Phytochemical-assisted green synthesis of CuFeOx nano-rose electrocatalysts for oxygen evolution reaction in alkaline media
Source: RSC Adv. 2023 Jun 23;13(28):19130–9. doi: 10.1039/d3ra02512h (PMC10288342; doi:10.1039/d3ra02512h)
Supplement: RA-013-D3RA02512H-s001 [file RA-013-D3RA02512H-s001.pdf]

Electronic Supplementary Material (ESI) for RSC Advances.
This journal is © The Royal Society of Chemistry 2023

Supplementary Information

Phytochemical-assisted green synthesis of $CuFeO_x$ nano-rose electrocatalysts for Oxygen Evolution Reaction in Alkaline Media

D. K Sarkar[a,b], V. Selvanathan[c*], M. Mottakin[a,d], A. K Mahmud Hasan[a], Md. Ariful Islam[a], Hamad Almohamadi[e*], Nabeel H. Alharthi[f,g] and Md. Akhtaruzzaman[a,h**]

a. Solar Energy Research Institute, Universiti Kebangsaan Malaysia, Bangi, Selangor Darul Ehsan 43600, Malaysia.

b. Department of Applied Chemistry and Chemical Engineering, Rajshahi University, Rajshahi-6205, Bangladesh.

c. Institute of Sustainable Energy, Universiti Tenaga Nasional (The Energy University), Jalan Ikram-Uniten, Kajang 43000, Selangor, Malaysia.

d. Department of Applied Chemistry and Chemical Engineering, Bangabandhu Sheikh Mujibur Rahman Science and Technology University, Gopalganj-8100, Bangladesh.

e. Department of Chemical Engineering, Faculty of Engineering, Islamic University of Madinah, Madinah, Saudi Arabia.

f. Department of Mechanical Engineering, Faculty of Engineering, Islamic University of Madinah, Madinah, Saudi Arabia.

g. Department of Mechanical Engineering, College of Engineering, King Saud University, Saudi Arabia, Riyadh, 11421, Saudi Arabia

h. Graduate School of Pure and Applied Sciences, University of Tsukuba, Tsukuba, Ibaraki 305-8573, Japan

*Corresponding Author: akhtar@ukm.edu.my; vidhya@ukm.edu.my; hha@iu.edu.sa

Energy dispersive X-ray (EDX):

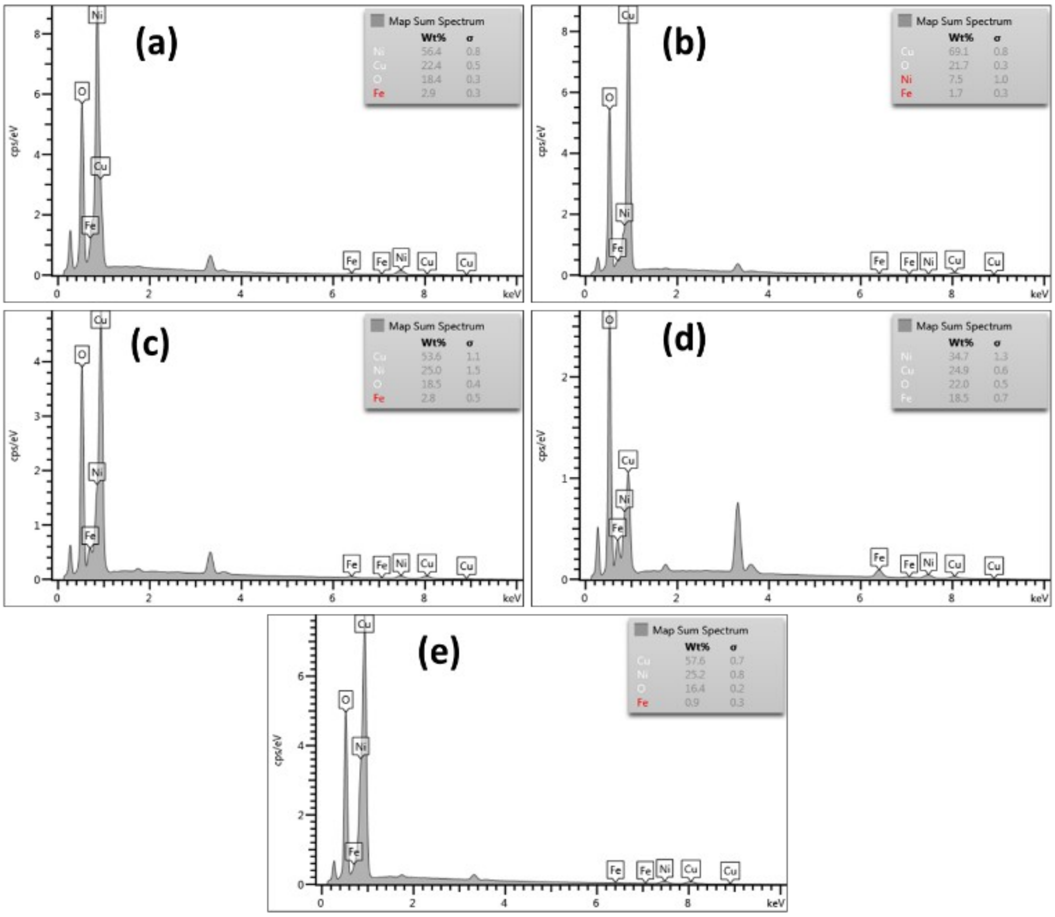

Figure S1: Elemental EDS analysis; (a): $CuFeO_x$–A, (b) $CuFeO_x$–B, (c) $CuFeO_x$–C, (d) $CuFeO_x$–D, (e) $CuFeO_x$–E.
